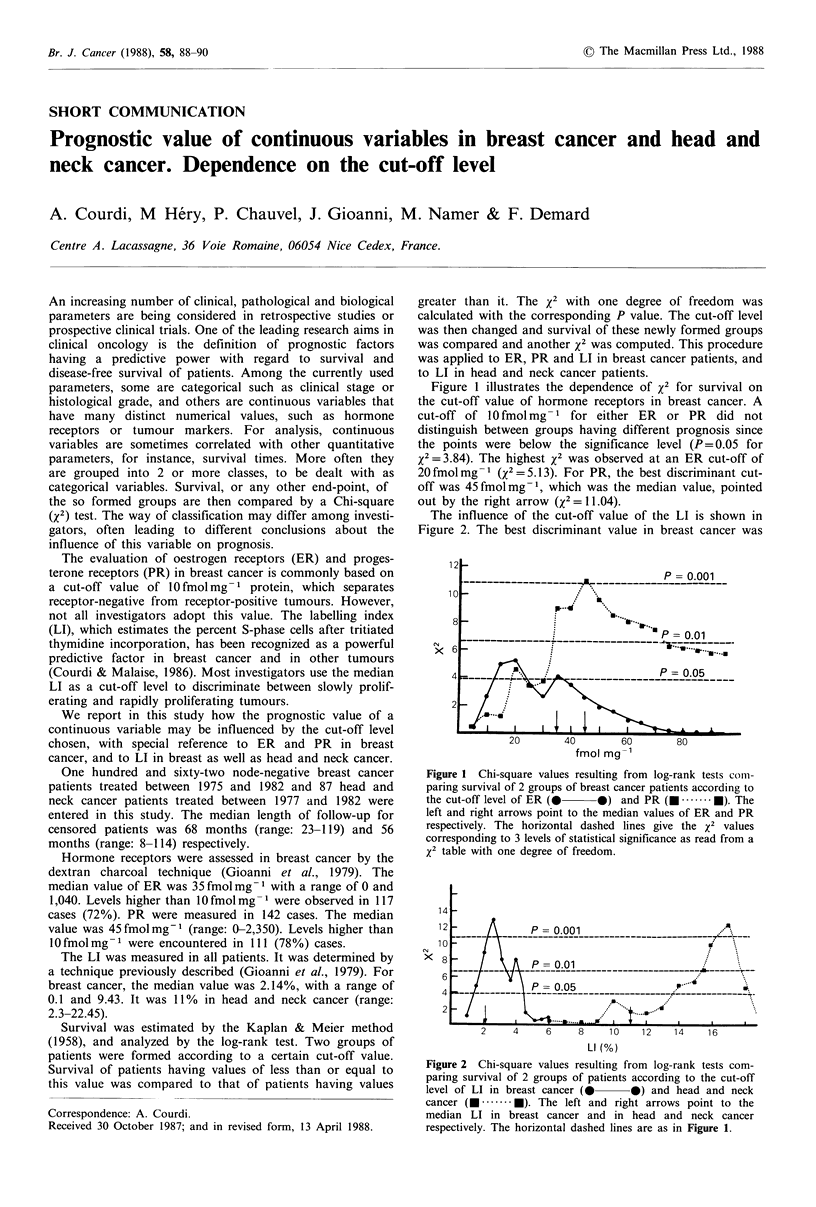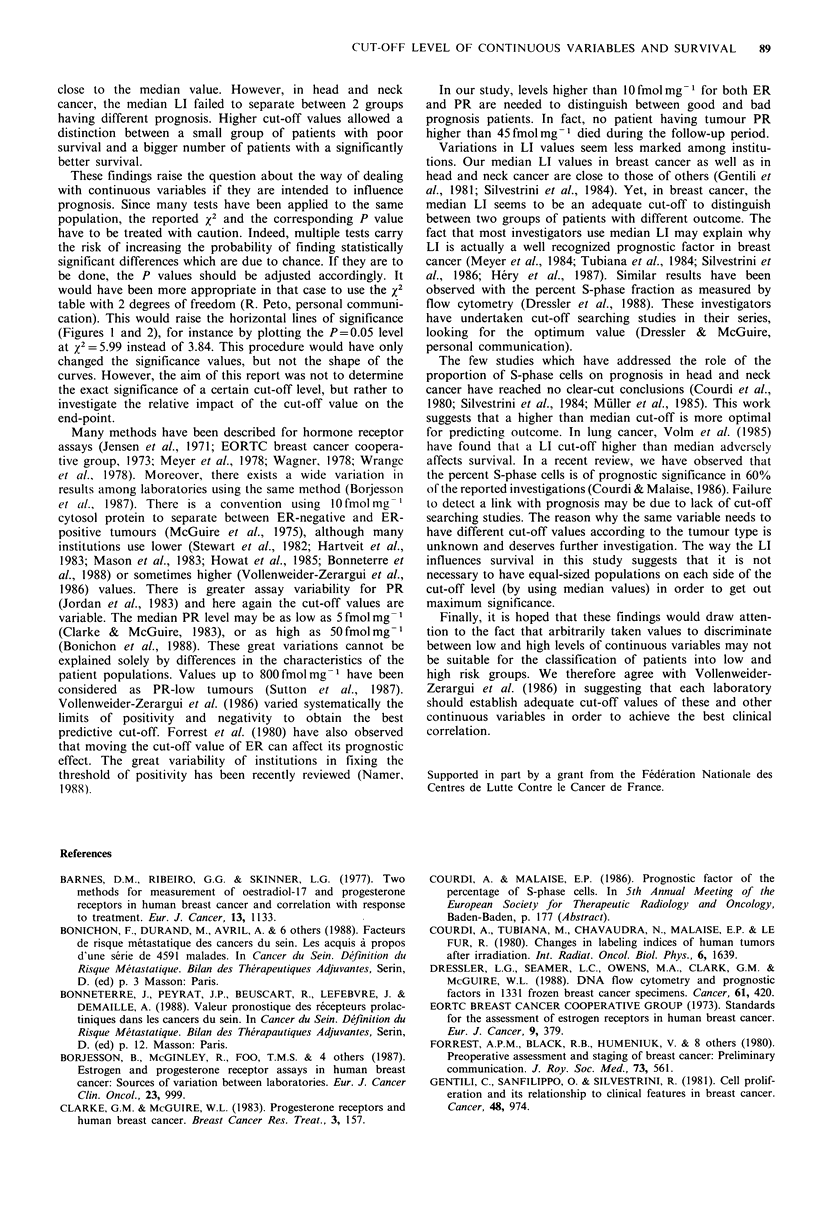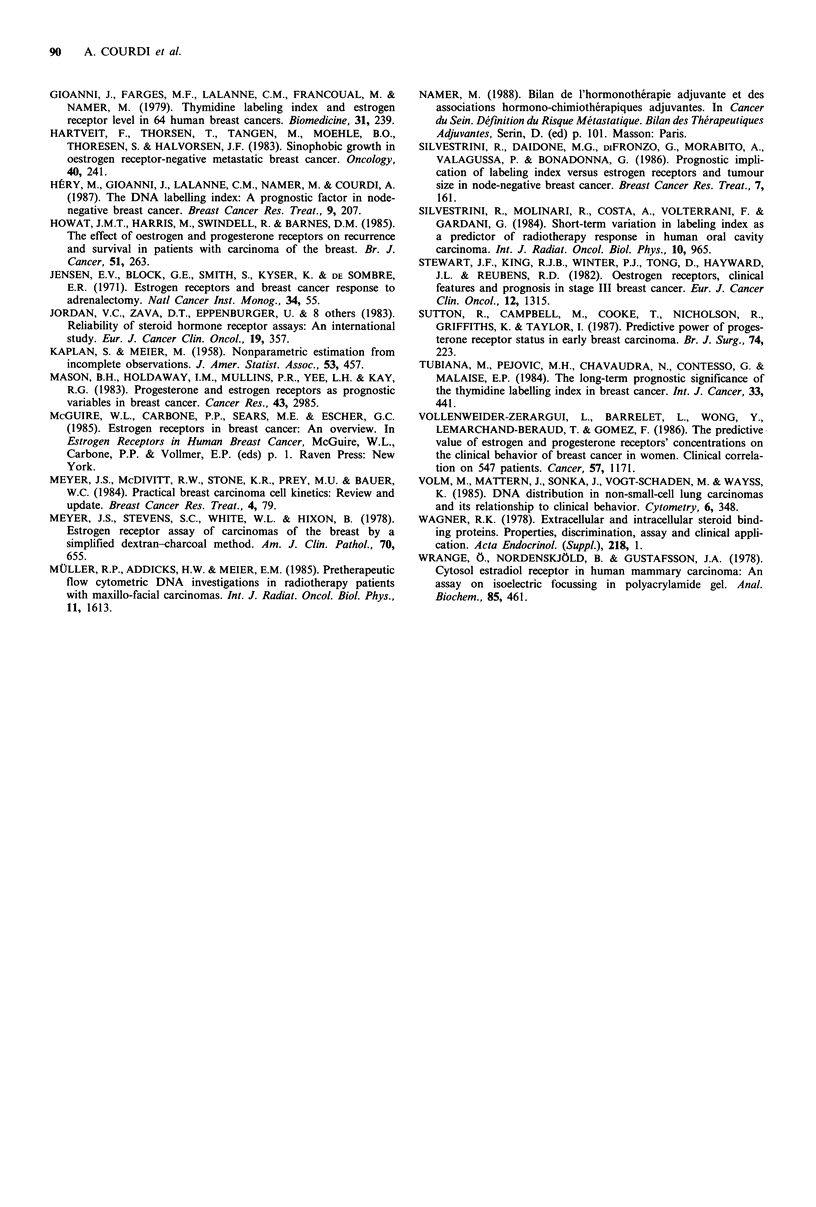# Prognostic value of continuous variables in breast cancer and head and neck cancer. Dependence on the cut-off level.

**DOI:** 10.1038/bjc.1988.169

**Published:** 1988-07

**Authors:** A. Courdi, M. HÃ©ry, P. Chauvel, J. Gioanni, M. Namer, F. Demard

**Affiliations:** Centre A. Lacassagne, Nice, France.


*Br. J. Cancer* (1988), **58**, 88–90 © The Macmillan Press Ltd., 1988

SHORT COMMUNICATION

### Prognostic value of continuous variables in breast cancer and head and neck cancer. Dependence on the cut-off level

A. Courdi, M Héry, P. Chauvel, J. Gioanni, M. Namer & F. Demard

*Centre A. Lacassagne, 36 Voie Romaine, 06054 Nice Cedex, France.*

An increasing number of clinical, pathological and biological parameters are being considered in retrospective studies or prospective clinical trials. One of the leading research aims in clinical oncology is the definition of prognostic factors having a predictive power with regard to survival and disease-free survival of patients. Among the currently used parameters, some are categorical such as clinical stage or histological grade, and others are continuous variables that have many distinct numerical values, such as hormone receptors or tumour markers. For analysis, continuous variables are sometimes correlated with other quantitative parameters, for instance, survival times. More often they are grouped into 2 or more classes, to be dealt with as categorical variables. Survival, or any other end-point, of the so formed groups are then compared by a Chi-square ($\chi^2$) test. The way of classification may differ among investigators, often leading to different conclusions about the influence of this variable on prognosis.

The evaluation of oestrogen receptors (ER) and progesterone receptors (PR) in breast cancer is commonly based on a cut-off value of $10\,\mathrm{fmol\,mg^{-1}}$ protein, which separates receptor-negative from receptor-positive tumours. However, not all investigators adopt this value. The labelling index (LI), which estimates the percent S-phase cells after tritiated thymidine incorporation, has been recognized as a powerful predictive factor in breast cancer and in other tumours (Courdi & Malaise, 1986). Most investigators use the median LI as a cut-off level to discriminate between slowly proliferating and rapidly proliferating tumours.

We report in this study how the prognostic value of a continuous variable may be influenced by the cut-off level chosen, with special reference to ER and PR in breast cancer, and to LI in breast as well as head and neck cancer.

One hundred and sixty-two node-negative breast cancer patients treated between 1975 and 1982 and 87 head and neck cancer patients treated between 1977 and 1982 were entered in this study. The median length of follow-up for censored patients was 68 months (range: 23–119) and 56 months (range: 8–114) respectively.

Hormone receptors were assessed in breast cancer by the dextran charcoal technique (Gioanni *et al.*, 1979). The median value of ER was $35\,\mathrm{fmol\,mg^{-1}}$ with a range of 0 and 1,040. Levels higher than $10\,\mathrm{fmol\,mg^{-1}}$ were observed in 117 cases (72%). PR were measured in 142 cases. The median value was $45\,\mathrm{fmol\,mg^{-1}}$ (range: 0–2,350). Levels higher than $10\,\mathrm{fmol\,mg^{-1}}$ were encountered in 111 (78%) cases.

The LI was measured in all patients. It was determined by a technique previously described (Gioanni *et al.*, 1979). For breast cancer, the median value was 2.14%, with a range of 0.1 and 9.43. It was 11% in head and neck cancer (range: 2.3–22.45).

Survival was estimated by the Kaplan & Meier method (1958), and analyzed by the log-rank test. Two groups of patients were formed according to a certain cut-off value. Survival of patients having values of less than or equal to this value was compared to that of patients having values greater than it. The $\chi^2$ with one degree of freedom was calculated with the corresponding $P$ value. The cut-off level was then changed and survival of these newly formed groups was compared and another $\chi^2$ was computed. This procedure was applied to ER, PR and LI in breast cancer patients, and to LI in head and neck cancer patients.

Figure 1 illustrates the dependence of $\chi^2$ for survival on the cut-off value of hormone receptors in breast cancer. A cut-off of $10\,\mathrm{fmol\,mg^{-1}}$ for either ER or PR did not distinguish between groups having different prognosis since the points were below the significance level ($P=0.05$ for $\chi^2=3.84$). The highest $\chi^2$ was observed at an ER cut-off of $20\,\mathrm{fmol\,mg^{-1}}$ ($\chi^2=5.13$). For PR, the best discriminant cut-off was $45\,\mathrm{fmol\,mg^{-1}}$, which was the median value, pointed out by the right arrow ($\chi^2=11.04$).

The influence of the cut-off value of the LI is shown in Figure 2. The best discriminant value in breast cancer was

**Figure 1** Chi-square values resulting from log-rank tests comparing survival of 2 groups of breast cancer patients according to the cut-off level of ER (●——●) and PR (■······■). The left and right arrows point to the median values of ER and PR respectively. The horizontal dashed lines give the $\chi^2$ values corresponding to 3 levels of statistical significance as read from a $\chi^2$ table with one degree of freedom.

**Figure 2** Chi-square values resulting from log-rank tests comparing survival of 2 groups of patients according to the cut-off level of LI in breast cancer (●——●) and head and neck cancer (■······■). The left and right arrows point to the median LI in breast cancer and in head and neck cancer respectively. The horizontal dashed lines are as in **Figure 1**.

Correspondence: A. Courdi.
Received 30 October 1987; and in revised form, 13 April 1988.

close to the median value. However, in head and neck cancer, the median LI failed to separate between 2 groups having different prognosis. Higher cut-off values allowed a distinction between a small group of patients with poor survival and a bigger number of patients with a significantly better survival.

These findings raise the question about the way of dealing with continuous variables if they are intended to influence prognosis. Since many tests have been applied to the same population, the reported $\chi^2$ and the corresponding $P$ value have to be treated with caution. Indeed, multiple tests carry the risk of increasing the probability of finding statistically significant differences which are due to chance. If they are to be done, the $P$ values should be adjusted accordingly. It would have been more appropriate in that case to use the $\chi^2$ table with 2 degrees of freedom (R. Peto, personal communication). This would raise the horizontal lines of significance (Figures 1 and 2), for instance by plotting the $P = 0.05$ level at $\chi^2 = 5.99$ instead of 3.84. This procedure would have only changed the significance values, but not the shape of the curves. However, the aim of this report was not to determine the exact significance of a certain cut-off level, but rather to investigate the relative impact of the cut-off value on the end-point.

Many methods have been described for hormone receptor assays (Jensen *et al.*, 1971; EORTC breast cancer cooperative group, 1973; Meyer *et al.*, 1978; Wagner, 1978; Wrange *et al.*, 1978). Moreover, there exists a wide variation in results among laboratories using the same method (Borjesson *et al.*, 1987). There is a convention using 10 fmol mg$^{-1}$ cytosol protein to separate between ER-negative and ER-positive tumours (McGuire *et al.*, 1975), although many institutions use lower (Stewart *et al.*, 1982; Hartveit *et al.*, 1983; Mason *et al.*, 1983; Howat *et al.*, 1985; Bonneterre *et al.*, 1988) or sometimes higher (Vollenweider-Zerargui *et al.*, 1986) values. There is greater assay variability for PR (Jordan *et al.*, 1983) and here again the cut-off values are variable. The median PR level may be as low as 5 fmol mg$^{-1}$ (Clarke & McGuire, 1983), or as high as 50 fmol mg$^{-1}$ (Bonichon *et al.*, 1988). These great variations cannot be explained solely by differences in the characteristics of the patient populations. Values up to 800 fmol mg$^{-1}$ have been considered as PR-low tumours (Sutton *et al.*, 1987). Vollenweider-Zerargui *et al.* (1986) varied systematically the limits of positivity and negativity to obtain the best predictive cut-off. Forrest *et al.* (1980) have also observed that moving the cut-off value of ER can affect its prognostic effect. The great variability of institutions in fixing the threshold of positivity has been recently reviewed (Namer, 1988).

In our study, levels higher than 10 fmol mg$^{-1}$ for both ER and PR are needed to distinguish between good and bad prognosis patients. In fact, no patient having tumour PR higher than 45 fmol mg$^{-1}$ died during the follow-up period.

Variations in LI values seem less marked among institutions. Our median LI values in breast cancer as well as in head and neck cancer are close to those of others (Gentili *et al.*, 1981; Silvestrini *et al.*, 1984). Yet, in breast cancer, the median LI seems to be an adequate cut-off to distinguish between two groups of patients with different outcome. The fact that most investigators use median LI may explain why LI is actually a well recognized prognostic factor in breast cancer (Meyer *et al.*, 1984; Tubiana *et al.*, 1984; Silvestrini *et al.*, 1986; Héry *et al.*, 1987). Similar results have been observed with the percent S-phase fraction as measured by flow cytometry (Dressler *et al.*, 1988). These investigators have undertaken cut-off searching studies in their series, looking for the optimum value (Dressler & McGuire, personal communication).

The few studies which have addressed the role of the proportion of S-phase cells on prognosis in head and neck cancer have reached no clear-cut conclusions (Courdi *et al.*, 1980; Silvestrini *et al.*, 1984; Müller *et al.*, 1985). This work suggests that a higher than median cut-off is more optimal for predicting outcome. In lung cancer, Volm *et al.* (1985) have found that a LI cut-off higher than median adversely affects survival. In a recent review, we have observed that the percent S-phase cells is of prognostic significance in 60% of the reported investigations (Courdi & Malaise, 1986). Failure to detect a link with prognosis may be due to lack of cut-off searching studies. The reason why the same variable needs to have different cut-off values according to the tumour type is unknown and deserves further investigation. The way the LI influences survival in this study suggests that it is not necessary to have equal-sized populations on each side of the cut-off level (by using median values) in order to get out maximum significance.

Finally, it is hoped that these findings would draw attention to the fact that arbitrarily taken values to discriminate between low and high levels of continuous variables may not be suitable for the classification of patients into low and high risk groups. We therefore agree with Vollenweider-Zerargui *et al.* (1986) in suggesting that each laboratory should establish adequate cut-off values of these and other continuous variables in order to achieve the best clinical correlation.

Supported in part by a grant from the Fédération Nationale des Centres de Lutte Contre le Cancer de France.

### References

BARNES, D.M., RIBEIRO, G.G. & SKINNER, L.G. (1977). Two methods for measurement of oestradiol-17 and progesterone receptors in human breast cancer and correlation with response to treatment. *Eur. J. Cancer*, **13**, 1133.

BONICHON, F., DURAND, M., AVRIL, A. & 6 others (1988). Facteurs de risque métastatique des cancers du sein. Les acquis à propos d'une série de 4591 malades. In *Cancer du Sein. Définition du Risque Métastatique. Bilan des Thérapeutiques Adjuvantes*, Serin, D. (ed) p. 3 Masson: Paris.

BONNETERRE, J., PEYRAT, J.P., BEUSCART, R., LEFEBVRE, J. & DEMAILLE, A. (1988). Valeur pronostique des récepteurs prolactiniques dans les cancers du sein. In *Cancer du Sein. Définition du Risque Métastatique. Bilan des Thérapautiques Adjuvantes*, Serin, D. (ed) p. 12. Masson: Paris.

BORJESSON, B., McGINLEY, R., FOO, T.M.S. & 4 others (1987). Estrogen and progesterone receptor assays in human breast cancer: Sources of variation between laboratories. *Eur. J. Cancer Clin. Oncol.*, **23**, 999.

CLARKE, G.M. & McGUIRE, W.L. (1983). Progesterone receptors and human breast cancer. *Breast Cancer Res. Treat.*, **3**, 157.

COURDI, A. & MALAISE, E.P. (1986). Prognostic factor of the percentage of S-phase cells. In *5th Annual Meeting of the European Society for Therapeutic Radiology and Oncology*, Baden-Baden, p. 177 (*Abstract*).

COURDI, A., TUBIANA, M., CHAVAUDRA, N., MALAISE, E.P. & LE FUR, R. (1980). Changes in labeling indices of human tumors after irradiation. *Int. Radiat. Oncol. Biol. Phys.*, **6**, 1639.

DRESSLER, L.G., SEAMER, L.C., OWENS, M.A., CLARK, G.M. & McGUIRE, W.L. (1988). DNA flow cytometry and prognostic factors in 1331 frozen breast cancer specimens. *Cancer*, **61**, 420.

EORTC BREAST CANCER COOPERATIVE GROUP (1973). Standards for the assessment of estrogen receptors in human breast cancer. *Eur. J. Cancer*, **9**, 379.

FORREST, A.P.M., BLACK, R.B., HUMENIUK, V. & 8 others (1980). Preoperative assessment and staging of breast cancer: Preliminary communication. *J. Roy. Soc. Med.*, **73**, 561.

GENTILI, C., SANFILIPPO, O. & SILVESTRINI, R. (1981). Cell proliferation and its relationship to clinical features in breast cancer. *Cancer*, **48**, 974.

GIOANNI, J., FARGES, M.F., LALANNE, C.M., FRANCOUAL, M. & NAMER, M. (1979). Thymidine labeling index and estrogen receptor level in 64 human breast cancers. *Biomedicine*, **31**, 239.

HARTVEIT, F., THORSEN, T., TANGEN, M., MOEHLE, B.O., THORESEN, S. & HALVORSEN, J.F. (1983). Sinophobic growth in oestrogen receptor-negative metastatic breast cancer. *Oncology*, **40**, 241.

HÉRY, M., GIOANNI, J., LALANNE, C.M., NAMER, M. & COURDI, A. (1987). The DNA labelling index: A prognostic factor in node-negative breast cancer. *Breast Cancer Res. Treat.*, **9**, 207.

HOWAT, J.M.T., HARRIS, M., SWINDELL, R. & BARNES, D.M. (1985). The effect of oestrogen and progesterone receptors on recurrence and survival in patients with carcinoma of the breast. *Br. J. Cancer*, **51**, 263.

JENSEN, E.V., BLOCK, G.E., SMITH, S., KYSER, K. & DE SOMBRE, E.R. (1971). Estrogen receptors and breast cancer response to adrenalectomy. *Natl Cancer Inst. Monog.*, **34**, 55.

JORDAN, V.C., ZAVA, D.T., EPPENBURGER, U. & 8 others (1983). Reliability of steroid hormone receptor assays: An international study. *Eur. J. Cancer Clin. Oncol.*, **19**, 357.

KAPLAN, S. & MEIER, M. (1958). Nonparametric estimation from incomplete observations. *J. Amer. Statist. Assoc.*, **53**, 457.

MASON, B.H., HOLDAWAY, I.M., MULLINS, P.R., YEE, L.H. & KAY, R.G. (1983). Progesterone and estrogen receptors as prognostic variables in breast cancer. *Cancer Res.*, **43**, 2985.

McGUIRE, W.L., CARBONE, P.P., SEARS, M.E. & ESCHER, G.C. (1985). Estrogen receptors in breast cancer: An overview. In *Estrogen Receptors in Human Breast Cancer*, McGuire, W.L., Carbone, P.P. & Vollmer, E.P. (eds) p. 1. Raven Press: New York.

MEYER, J.S., McDIVITT, R.W., STONE, K.R., PREY, M.U. & BAUER, W.C. (1984). Practical breast carcinoma cell kinetics: Review and update. *Breast Cancer Res. Treat.*, **4**, 79.

MEYER, J.S., STEVENS, S.C., WHITE, W.L. & HIXON, B. (1978). Estrogen receptor assay of carcinomas of the breast by a simplified dextran–charcoal method. *Am. J. Clin. Pathol.*, **70**, 655.

MÜLLER, R.P., ADDICKS, H.W. & MEIER, E.M. (1985). Pretherapeutic flow cytometric DNA investigations in radiotherapy patients with maxillo-facial carcinomas. *Int. J. Radiat. Oncol. Biol. Phys.*, **11**, 1613.

NAMER, M. (1988). Bilan de l'hormonothérapie adjuvante et des associations hormono-chimiothérapiques adjuvantes. In *Cancer du Sein. Définition du Risque Métastatique. Bilan des Thérapeutiques Adjuvantes*, Serin, D. (ed) p. 101. Masson: Paris.

SILVESTRINI, R., DAIDONE, M.G., DIFRONZO, G., MORABITO, A., VALAGUSSA, P. & BONADONNA, G. (1986). Prognostic implication of labeling index versus estrogen receptors and tumour size in node-negative breast cancer. *Breast Cancer Res. Treat.*, **7**, 161.

SILVESTRINI, R., MOLINARI, R., COSTA, A., VOLTERRANI, F. & GARDANI, G. (1984). Short-term variation in labeling index as a predictor of radiotherapy response in human oral cavity carcinoma. *Int. J. Radiat. Oncol. Biol. Phys.*, **10**, 965.

STEWART, J.F., KING, R.J.B., WINTER, P.J., TONG, D., HAYWARD, J.L. & REUBENS, R.D. (1982). Oestrogen receptors, clinical features and prognosis in stage III breast cancer. *Eur. J. Cancer Clin. Oncol.*, **12**, 1315.

SUTTON, R., CAMPBELL, M., COOKE, T., NICHOLSON, R., GRIFFITHS, K. & TAYLOR, I. (1987). Predictive power of progesterone receptor status in early breast carcinoma. *Br. J. Surg.*, **74**, 223.

TUBIANA, M., PEJOVIC, M.H., CHAVAUDRA, N., CONTESSO, G. & MALAISE, E.P. (1984). The long-term prognostic significance of the thymidine labelling index in breast cancer. *Int. J. Cancer*, **33**, 441.

VOLLENWEIDER-ZERARGUI, L., BARRELET, L., WONG, Y., LEMARCHAND-BERAUD, T. & GOMEZ, F. (1986). The predictive value of estrogen and progesterone receptors' concentrations on the clinical behavior of breast cancer in women. Clinical correlation on 547 patients. *Cancer*, **57**, 1171.

VOLM, M., MATTERN, J., SONKA, J., VOGT-SCHADEN, M. & WAYSS, K. (1985). DNA distribution in non-small-cell lung carcinomas and its relationship to clinical behavior. *Cytometry*, **6**, 348.

WAGNER, R.K. (1978). Extracellular and intracellular steroid binding proteins. Properties, discrimination, assay and clinical application. *Acta Endocrinol.* (*Suppl.*), **218**, 1.

WRANGE, Ö., NORDENSKJÖLD, B. & GUSTAFSSON, J.A. (1978). Cytosol estradiol receptor in human mammary carcinoma: An assay on isoelectric focussing in polyacrylamide gel. *Anal. Biochem.*, **85**, 461.